# Mizaj as an Index in Persian Traditional Medicine Index Could Associate with Sensitivity to the Radiation

**DOI:** 10.31661/gmj.v9i0.1705

**Published:** 2020-08-19

**Authors:** Fatemeh Asghari, Kourosh Ebrahimnejad Gorji, Seyyed Ali Mozaffarpur, Ali Shabestani Monfared, Ebrahim Zabihi, Zeinab Abedian, Hoda Shirafkan, Fatemeh Niksirat, Sajad Borzoueisileh

**Affiliations:** ^1^Student Research Committee, Babol University of Medical Sciences, Babol, Iran; ^2^Department of Medical Physics Radiobiology and Radiation Protection, School of Medicine, Babol University of Medical Sciences, Babol, Iran; ^3^Traditional Medicine and History of Medical Sciences Research Center, Health Research Institute, Babol University of Medical Sciences, Babol, Iran; ^4^Cancer Research Center, Health Research Institute, Babol University of Medical Sciences, Babol, Iran; ^5^Cellular and Molecular Biology Research Center, Health Research Institute, Babol University of Medical Sciences, Babol, Iran; ^6^Social Determinants of Health Research Center, Health Research Institute, Babol University of Medical Sciences, Babol, Iran

**Keywords:** Complementary Therapies, Comet Assay, Radiation Tolerance, Traditional, Mizaj

## Abstract

**Background:**

The sensitivity to the radiation among human population depends on various parameters. This variation could lead to dissimilar outcome of radiotherapy in similar situations. Mizaj is a well-known term in Persian medicine that present an individualized medicine viewpoint. All of the people will be categorized in cold, moderate, and warm Mizaj. In this study, we aimed to evaluate the possible association between Mizaj and radiosensitivity by comet assay.

**Materials and Methods:**

Peripheral blood sample of 30 healthy volunteers (10 cold, 11 moderate and nine warm Mizaj) were taken and divided into two identical parts. The first part was exposed to 4 Gy x-rays, and the second part was regarded as the sham control. Then, DNA damages of samples were evaluated by the neutral comet assay.

**Results:**

The results showed that the mean percentage of damaged cells, in all of the irradiated groups including A (warm), B (moderate) and C (cold) was significantly higher than the controls (P<0.001). Moreover, DNA damage rate in the irradiated warm Mizaj group was higher than both cold and moderate irradiated groups, but the difference between moderate and cold irradiated groups was not statistically significant.

**Conclusion:**

The results are indicating that warm Mizaj persons could be more radiosensitive than other groups, which their importance in radiotherapy individualization should be evaluated in more extensive studies.

## Introduction

Radiosensitivity is relative vulnerability to the dangerous effects of ionizing radiation. The response to radiation depends on many factors such as radiation type, energy, and dose rate. Also. The biological and chemical properties of cells or tissues could affect the response [1] then, similar irradiation condition may induce different reactions to radiation in different patients [2]. Genetic is one of the most important biological factors which could affect the radiation sensitivity [3]. The results of studies have shown that the genes are involved in DNA repairs, such as ATM and NBS have an essential role in the biological response to radiation. It is shown that mutations in the ataxia-telangiectasia gene could alter the response to radiation [4]. Also, a study of radiosensitivity in head and neck cancers have reported that differential expression of ku80 gene can be a predictive factor for radiosensitivity [5]. The correlation between some SNPs and cellular response to radiation has investigated too [6,7]. Differences in genome sequencing and gene expression cause various phenotypes, and these differences can potentially affect the risk of diseases and responses to therapies or drugs [8]. This difference is the basic theory of personalized medicine, a relatively new medical approach that was based on differences among peoples. The concept of Mizaj is considered as a principle in many traditional medicine cultures. In traditional Persian medicine (PM), an individualized viewpoint is introduced by the term of Mizaj. The root of Mizaj (hot and cold natures), is from ancient Greece (Hippocrates, a Greek physician, 460-375). The most critical activity in this area in the east was carried out by Avicenna (Persian physician, 937-1037), who tried to collect and organize all the ancient theories and mixed them with the medical approaches of the time and wrote the book “Canon of medicine”. The maintenance of the equilibrium between the fundamental body elements was the most important principle of the book. Ten criteria focused on physical, physiological, and mental status are propose for Mizaj assessment in PM [9]. Some studies showed that some disorders are related to Mizaj. The type of Mizaj can be responsible for the risk of some diseases [10]. Also, Mizaj can alter the response to treatment, e.g., it was reported that some drugs are more efficient for cold Mizaj persons, another study described an association between blood groups and types of Mizaj [11]. Consequently, as the theory of Mizaj in PM is a sort of individualized viewpoints in medicine, it can theoretically cause different radiosensitivity in persons. Comet assay is a cytogenetic method that directly detects DNA damage such as single-strand break (SSB) and double-strand break (DSB) and also repair. The assay is relatively cheap, rapid, and easy to work. It does not need cell culture, and the results could be obtained within hours after sampling [12]. The assay is previously used for the study of radiosensitivity in human cells [13]. The possible association between Mizaj and radiosensitivity was not studied yet; then, the present study aimed to compare radiosensitivity in warm and cold Mizaj persons by the neutral comet assay.

## Materials and Methods

###  Objective and Study Design

 In this case-control study, we have evaluated the association between radiation-induced comet formation rate (as a technique for evaluating of radiosensitivity) and Mizaj in peripheral blood lymphocytes, based on PM theories. The study duration was 26 weeks started from 28 April up to 7 December 2016, and it was approved by Ethical Committee of Babol University of Medical Sciences, Babol, Iran.

###  Study Population and Recruitment Strategy 

 Individuals who fulfilled the following criteria entered into the study: Twenty to 40 years old men or women, general good health, without any known disease and continues the use of drugs. Handedness and blood group had a relationship with radiosensitivity, so we selected right-hander and O blood group individuals to control the effects of these factors. The exclusion criteria were the history of radiation therapy, radiation workers, smoking, alcohol use, and pregnancy.

###  Screening Process 

**Figure 1:**
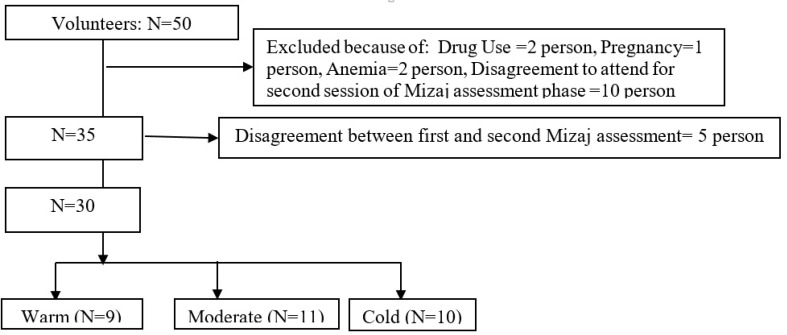
Mizaj assessment process.

 Potential participants were subjected to visit by physicians to assess if they met the eligibility criteria. Fifty-two participants completed a screening questionnaire. The procedure of the study was explained, and the written informed consents were completed, then they attended the study (Figure-1).

###  1.Study Intervention

####  1. 2.Mizaj assessment

 Three experts in PM (MD. Ph.D.) With at least five years of clinical practice, visited all volunteers in 2 different sessions with at least two weeks gap. Mizaj assessment was done separately for each participant and recorded on a sheet; then an expert panel discussion was done to finalize the diagnosis. Thirty entered individuals were categorized in 3 Mizaj of warm, moderate, and cold.

####  1. 3.Blood sampling and irradiation 

 Two-milliliter blood samples of the volunteers were taken under the sterile condition and collected in micro-tubes containing sodium heparin anticoagulant. The samples were divided into two equal values (1 ml) and kept in similar conditions. One part of each sample was exposed to 4 Gy (200 cGy per min, SSD=100 cm) of x-rays from Linac (ELEKTA Compact 6MV). The second part, regarded as the control.

###  Neutral Comet Assay

 After irradiations, the DNA damages were evaluated by the neutral comet assay. Briefly, the samples were centrifuged at 2500 rpm for 5 min, and the remainder (10µL) was mixed with 140 µL of 0.75% low melting point agarose (Fermentaz, Poland) dissolved in distilled water. Then, 50 µL of the suspension was applied to each window slides coated with a layer of 1% normal melting point agarose (Fermentaz, Poland). The slides were covered and solidified in 5 min. Then, the slides were submersed within 30 minutes at 4 ºc in lysis solution, which consist of (2.5 M NaCl (Merck, Germany), 0.1 M Na2EDTA (Merck, Germany), 10 mM Tris-base (Merck, Germany), % Triton X-100(SIGMA, USA), 10% dimethyl sulfoxide (Merck, Germany), PH=10). After lysing, the slides were washed with electrophoresis buffer (90 mM Tris base, 90 mM Boric acid and 2.5 mM Na2EDTA, PH=8.3-8.4) for 15 min at 4 ºc. Afterward, the slides were located in a horizontal electrophoresis chamber with the fresh electrophoresis buffer, for 15 min at 20 v and 9 mA. Then, Slides were washed with distilled water for 5 min, rinsed in 96% ethanol for 5 min and dried at room temperature. In the end, slides were stained with ethidium bromide (20 µL/ml) Merck, Germany).

###  Microscopic Analysis 

The slides were analyzed with a fluorescent microscope (E800, Nikon, Japan) with Wavelength (WL) 590 nm and magnification power 200. The slides were analyzed by visual detection and classified into 4 grade (N1-N4) base on the length of the comet tail that shows the severity of DNA damage [14]. The total damages were calculated using the following equation:

 DD=(0N0+1N1+2N3+3N3+4N4)/ (∑/100)

 DD=DNA Damage

 N=Number of comet

 ∑: Total number of comets

###  Statistical Analysis

 The data were analyzed by SPSS 14 software (SPSS for Windows, SPSS, Chicago, USA), and the normality was checked by the Kolmogorov-Smirnov test. The analyses were performed by paired t-test between the control and exposed groups and ANOVA with significance accepted at the 5% level were used to compare the DNA damage between three exposed groups of Mizaj. The demographic characteristics were described by descriptive statistics, including mean ± standard deviations, frequency, and percentage. The error bars were applied by 95% confidence interval.

## Results

**Figure 2:**
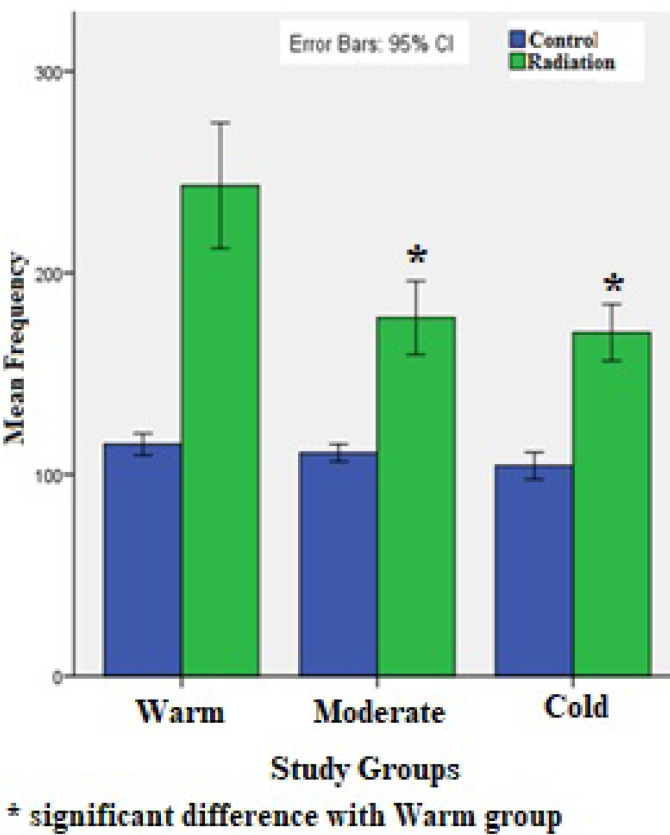
Mean frequency of damaged cells in control and irradiated groups of different Mizaj.

**Figure 3:**
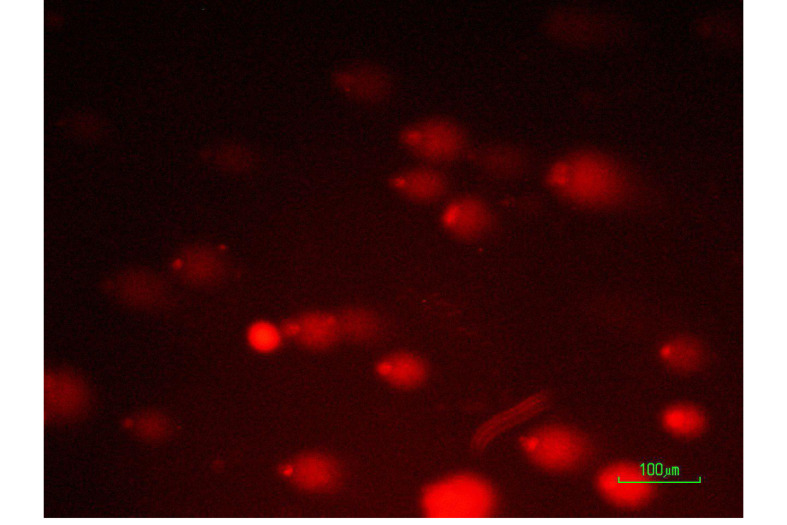
A photomicrograph of the comet.

 After considering the inclusion and exclusion criteria, 30 out of 50 volunteers entered into the study. The Mizaj assessment process is shown in Figure-1. The Demographic characteristics, including age, height, weight, and body mass index (BMI) of the included population were shown in Table-1. The Mean frequency of damaged cells in blood samples exposed to 4 Gy irradiation were shown in Figure-1. The results showed that warm Mizaj persons have the highest radiosensitivity (P<0.001) but the difference between moderate (177.73±27.13) and cold Mizaj (170.40±19.66) groups was not significant (P=0.84). According to Figure-2, the mean percentage of damaged cells, in the irradiated samples for all of the groups including warm, moderate, and cold Mizaj was significantly higher than the controls (P<0.001). The increase of damages after exposure to 4 Gy x-rays for all of the three groups was significant. A photomicrograph of our comet assay results were shown in Figure-3.

## Discussion

The idea of the difference in radiosensitivity among individual’s Mizaj characters is proposed. 30 entered individuals were categorized in 3 Mizaj of warm, moderate, cold, and the rate of DNA damages were compared after x-ray irradiation. We have applied an expert panel to determine the Mizaj. The Mizaj assessment was performed in two separate sessions to authenticate the result to enhance its validity. Disagreement in two sessions was excluded from the study. The most interesting finding of our study indicated that the number of DNA damage in the warm Mizaj group is significantly higher than the cold Mizaj individuals (P-value<0.001). Based on the traditional PM, the Mizaj varies with age, and we selected healthy young volunteers to enroll in the study. In this study, warm groups had more instability in DNA than moderate and cold Mizaj. Although there was a difference between radiosensitivity of two groups of cold and moderate Mizaj persons, it was not statistically significant. This result may be due to the Mizaj assessment protocol that results in more nearness between moderate and cold Mizaj groups than the similarities between moderate and warm Mizaj ones. In this study, radiation exposure induced significant DNA damage in agreement with studies of Koksal *et al*. [15]. The radiation-induced DNA damage is a fact, and we were not surprised by this outcome. A clinical study of radiosensitivity in breast cancer patients has shown the different reactions of patients to the same dose and reported that some patients displayed severe reactions to radiation therapy [2]. According to the present study, warm Mizaj persons shown higher radiosensitivity but the difference between moderate Mizaj and cold Mizaj groups was not significant. Based on Traditional medicine, Mizaj is an inherent trait that is identified with physical, physiological, and psychological differences among people and as a result, different criteria have been proposed for Mizaj assessment based on PM. Shahabi *et al*. reported that warm nature individuals have a more sympathetic activity. They also cited that their Th2 immune system responses are more often [16]. Another study has declared high frequency of allergic reactions exist in warm Mizaj persons [17]. Investigation of Cheng A *et al*. displayed that the efficiency of anti-inflammatory drugs in patients with a cold pattern is higher than those with a warm pattern [18]. A previous study performed by Rashid Bikha *et al*. indicated that some disorders such as diabetes and hypertension are more common in persons with warm Mizaj [10]. Also, it is suggested that warm Mizaj persons are more prone to disorders such as acute fever, stroke, dehydration. The study performed by Jongmans W *et al*. described an association between genes such as CABLES1, WWOX and IFI27 and warm Mizaj persons with RA [18]. Thus, Mizaj, as an individualized medicine indicator, can be used for predicting the radiosensitivity. Based on PM theories, warmness can cause instability, so it supposed that warm individuals have more movement, with high temperament, various and rapid reactions to external stimulators. Although these reactions were not surveyed yet and in the case of approval, it could be generalized to the level of cells, and our findings can confirm this theory. It means that warmness of individuals widespread in their cells and shows the same reaction that we suppose to see in general reaction of a person. Lake of a reliable and valid standard questionnaire of Mizaj is a limitation of our study. We tried to eliminate this defect with two session assessment of Mizaj based on an expert panel opinion that is the best available gold standard. The low sample size is another limitation of our study, but the results have been significantly based on the interim analysis. More studies can be suggested with different tests of radiosensitivity and with higher sample size. As it is proposed in previous studies, some genes such as ATM and NBS, have roles in DNA repair, surveying the correlation between the existence of these genes and Mizaj of individuals, can be the matter of future studies.

## Conclusion

 In conclusion, the results are indicating that warm Mizaj persons could be more radiosensitive than other groups, which their importance in radiotherapy individualization should be evaluated in extensive studies.

## Conflict of Interest

 The authors declare no conflicts of interest.

**Table 1:** the demographic characteristics of different study groups including age, height, weight, and body mass index (BMI) of the included population.

**Variables**	**Warm (Mean ±SD)**	**Moderate (Mean ±SD)**	**Cold (Mean ±SD)**	**P-value**
**age**	29±5	25±6	27±4	0.299
**height**	167±9	166±7	163±5	0.533
**weight**	68±20	63±12	58±10	0.370
**BMI**	23.95±4.99	22.82±3.33	21.84±2.99	0.493
